# Supplementary material for: Arabinofuranosyl Thymine Derivatives—Potential Candidates against Cowpox Virus: A Computational Screening Study
Source: Int J Mol Sci. 2023 Jan 16;24(2):1751. doi: 10.3390/ijms24021751 (PMC9864678; doi:10.3390/ijms24021751)
Supplement: Supplementary file 1 [file ijms-24-01751-s001.zip › ijms-2149330-supplementary.pdf]

Table S1. Thymine-related structures were used in the study.

**Structures**	**PubChem ID**
**Cidofovir (Standard)**	60613
**(1)**	65049
**(2)**	11243988
**(3)**	123370001
**(4)**	129802452
**(5)**	154137224
**(6)**	191372
**(7)**	21308117
**(8)**	451892
**(9)**	59119818
**(10)**	90413362
**(11)**	90413364
**(12)**	1135
**(13)**	566009
**(14)**	78957
**(15)**	667607
**(16)**	330104
**(17)**	78112
**(18)**	3385
**(19)**	348851
**(20)**	69672
**(21)**	163114065

Table S2. Generated pharmacophore models with scores

Pharmacophore models	Score (0-1)
Model-1	0.8320
Model-2	0.8163
Model-3	0.8064
Model-4	0.8033
Model-5	0.7995
Model-6	0.7994
Model-7	0.7909
Model-8	0.7908
Model-9	0.7850
Model-10	0.7722
